# Harnessing the power of traditional Chinese medicine monomers and compound prescriptions to boost cancer immunotherapy

**DOI:** 10.3389/fimmu.2023.1277243

**Published:** 2023-11-15

**Authors:** Keyan Miao, Weici Liu, Jingtong Xu, Zhengtao Qian, Qinglin Zhang

**Affiliations:** ^1^Medical College, Soochow University, Suzhou, Jiangsu, China; ^2^Department of Thoracic Surgery, The Affiliated Wuxi People’s Hospital of Nanjing Medical University, Wuxi People’s Hospital, Wuxi Medical Center, Nanjing Medical University, Wuxi, Jiangsu, China; ^3^The First School of Clinical Medicine, Nanjing Medical University. Nanjing, Jiangsu, China; ^4^Department of Clinical Laboratory, Changshu Medicine Examination Institute, Changshu, Jiangsu, China; ^5^Department of Gastroenterology, The Affiliated Wuxi People’s Hospital of Nanjing Medical University, Wuxi People’s Hospital, Wuxi Medical Center, Nanjing Medical University, Wuxi, Jiangsu, China

**Keywords:** traditional Chinese medicine, natural products, tumor immunity, cancer immunotherapy, immunomodulators, cytokines

## Abstract

At present, cancer is the largest culprit that endangers human health. The current treatment options for cancer mainly include surgical resection, adjuvant radiotherapy and chemotherapy, but their therapeutic effects and long-term prognosis are unsatisfactory. Immunotherapy is an emerging therapy that has completely transformed the therapeutic landscape of advanced cancers, and has tried to occupy a place in the neoadjuvant therapy of resectable tumors. However, not all patients respond to immunotherapy due to the immunological and molecular features of the tumors. Traditional Chinese Medicine (TCM) provides a new perspective for cancer treatment and is considered to have the potential as promising anti-tumor drugs considering its immunoregulatory properties. This review concludes commonly used TCM monomers and compounds from the perspective of immune regulatory pathways, aiming to clearly introduce the basic mechanisms of TCM in boosting cancer immunotherapy and mechanisms of several common TCM. In addition, we also summarized closed and ongoing trials and presented prospects for future development. Due to the significant role of immunotherapy in the treatment of non-small cell lung cancer (NSCLC), TCM combined with immunotherapy should be emphasized in NSCLC.

## Highlights

- TCM is a promising cancer treatment with three primary advantages including fewer side effects, personalized treatment, and potential immune system benefits.
- TCM exerts an effect on tumor immunity through multiple modalities of regulation.
- The pertinent studies and mechanisms of several commonly utilized TCM active ingredients were preliminarily reported and unconcealed.
- Immunotherapy in combination with TCM selectively targets cancer cells for suppression, leaving normal cells unharmed.

## 1 Introduction

Due to its profound impact on public health, the malignant tumor has emerged as a pressing concern in society, characterized by substantial morbidity and mortality rates (1). Presently, surgery and chemotherapy stand as the primary modalities for cancer treatment (2). Despite decades of dedicated research efforts towards unearthing more efficacious approaches, the global incidence of malignant tumors continues to remain alarmingly high. The intricate and heterogeneous nature of tumors has prompted a paradigm shift in cancer management, transitioning from a singular target approach to a multifaceted strategy that encompasses the tumor microenvironment (TME) (2–4).

Tumor immunotherapy, which refers to a type of cancer therapies that aims to harness the natural ability of the immune system to identify and finally eliminate tumor cells, seems to display a promising future as a potential therapeutic option for various kinds of tumors (5, 6). This approach involves the application of various therapeutic agents and strategies that modulate or enhance immune responses against cancer, including agents that activate immune cells such as T cells, antibodies that target specific cancer cells, and cellular therapies that involve the transfer of genetically engineered T cells or other immune cells into cancer patients (7, 8). Unlike traditional cancer therapies such as chemotherapy and radiation, which target both cancerous and normal cells, tumor immunotherapy has an edge of selectively targeting and eliminating cancer cells and minimizing damage to normal tissues. Current drugs have a therapeutic effect on cancer in the short term, but their major side effects can cause serious physical and mental problems for patients. Therefore, there is an urgent need to explore new therapeutic methods that have definite efficacy and mild adverse effects and can be taken for a long time.

For centuries, TCM has been employed as a therapeutic approach for various ailments, with cancer included, boasting a track record of safety, gentleness, and prolonged efficacy (9, 10). TCM adopts a holistic perspective, considering tumors as systemic diseases where treatment should encompass the entire body rather than solely focusing on localized areas. Approaches limited to local therapies often fail to provide comprehensive clinical benefits to cancer patients, thereby contributing to the underlying factors responsible for tumor recurrence and metastasis even after surgical interventions, chemotherapy, or targeted therapies. The advantages of TCM in the tumor immunotherapy can be summarized into three aspects——fewer side effects, personalized treatment, and potential immune system benefits (11–13). Chinese herbal therapy typically takes advantage of natural substances that are less toxic and have fewer side effects compared to chemoradiotherapy drugs (11). Chemoradiotherapy can cause side effects such as nausea, vomiting, hair loss, and fatigue, whereas Chinese herbal therapy is generally well-tolerated and can improve quality of life (12, 13). Chinese herbal therapy is tailored to the individual patient’s needs and can be adjusted based on their response to treatment. Chemoradiotherapy, on the other hand, is a standardized treatment that is prescribed based on the type and stage of cancer, and may not consider the individual differences in patients’ overall health or response to treatment. Chinese herbal therapy can enhance the body’s natural immune system, which can effectively detect and eliminate cancer cells and prevent recurrence. Chemoradiotherapy can weaken the immune system, making the patient more vulnerable to infections and other complications (14, 15). Overall, while there is still much to learn about the potential of TCM for the treatment of tumor, research to date suggests that it may have a role to play in improving outcomes for cancer patients (12, 15).

Currently, research on immunotherapy methods is underway, however, the investigation of possible targets from the perspective of TCM is still in its infancy. Considering this, it is desperately demanded to explore the potential and development direction of TCM as an immunotherapy approach. We will discuss the specific monomers and compounds found in TCM that have been identified to have potential in boosting cancer immunotherapy, as well as the mechanisms by which they work. We will also explore the results of clinical studies that have investigated the application of TCM in combination with cancer immunotherapy, as well as the obstacles and future directions for research in this field.

## 2 The regulatory effects of TCM monomers and compounds on TME

**Figure 1:**
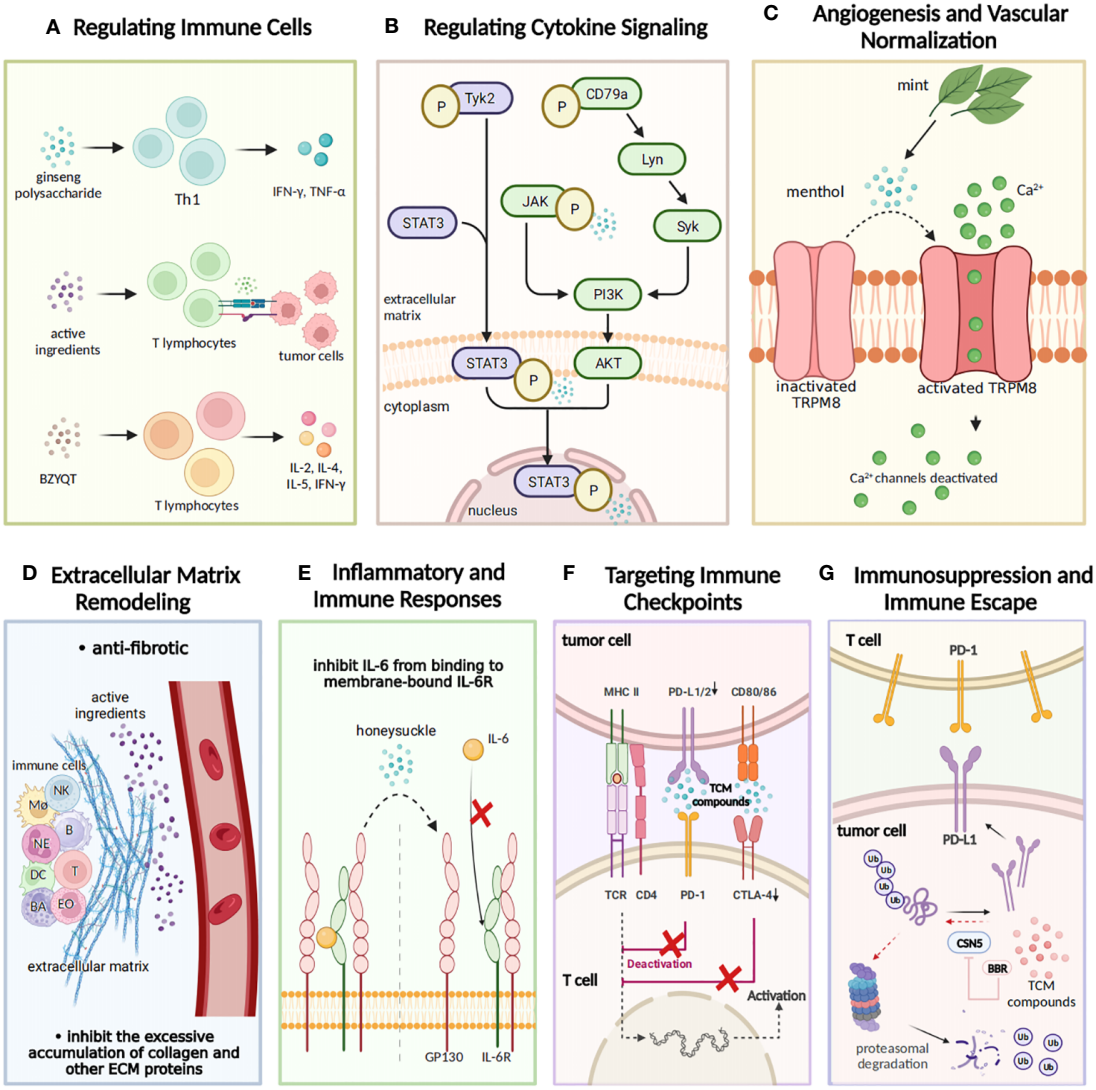
The regulatory effects of TCM on tumor immunity. TCM exerts a regulatory influence on tumor immunity from seven aspects. **(A)** Regulating immune cells: TCM exhibits immunomodulatory properties by elevating levels of IL-2, IL-8, and TNF-α and facilitating the recognition and binding of T lymphocytes to tumor cells. The TCM prescription Bu-Zhong-Yi-Qi-Tang (BZYQT) stimulates cytokine production from T lymphocytes including IL-2, IL-4, IL-5, and IFN-γ, thus demonstrating its immunomodulatory efficacy. **(B)** Modulating cytokine signaling: Gansui-Banxia Decoction (GSBXD), a classic TCM formula, exerts anti-tumor immune activity by downregulating AKT/STAT3/ERK signaling pathways and suppressing IL-1β and IFN-γ. **(C)** Angiogenesis and vascular normalization: Menthol activates angiogenesis by promoting the opening of TRPM8 ion channels and facilitating calcium ion flow. **(D)** Remodeling of extracellular matrix: TCM compounds regulate the intricate processes of extracellular matrix (ECM) remodeling, including degradation and deposition of ECM components. Additionally, they effectively inhibit excessive accumulation of collagen and other ECM proteins. **(E)** Inflammatory and immune responses: Honeysuckle inhibits the binding of IL-6 to membrane-bound IL-6R, thereby impeding tumor initiation and progression. **(F)** Targeting immune checkpoints: TCM compounds disrupt the binding of programmed cell death ligand 1 (PD-L1) and programmed cell death protein 1 (PD-1), impacting their inhibitory effect and promoting normal immune function. **(G)** Immunosuppression and immune escape: TCM compounds inhibit the expression of immune checkpoint molecules, such as PD-1 and cytotoxic T-lymphocyte associated protein 4 (CTLA-4), hence restoring immune cell functionality and facilitating robust tumor immune responses.

Immunomodulation means the ability of a substance to modify and regulate the immune response, either by enhancing or suppressing it. Chinese herbal medicine has been uncovered to have both immunostimulatory and immunosuppressive effects, depending on the specific herbs and the context in which they are applied (16–18). The immunoregulatory effects of Chinese medicine include the stimulation of immune cell activity, regulation of cytokine production, inhibition of inflammation, antioxidant activity, modulation of immune cell signaling pathways, etc (16, 19–21). TCM monomers such as baicalin, quercetin, and resveratrol have been reported to modulate the production of cytokines by regulating immune cells (22–25). These monomers can either enhance or suppress cytokine production, relying on the specific immune cells and the specific environment in which they are distributed (26). TCM monomers such as curcumin and berberine have been discovered to have anti-inflammatory effects through suppressing the production of pro-inflammatory cytokines and inhibiting the activation of immune cells such as macrophages. TCM monomers can regulate a variety of signaling pathways involved in immune cell activation and function. For instance, curcumin has been revealed to inhibit the nuclear factor-kappa B (NF-kB) signaling pathway, which plays a crucial role in regulating immune cell activity and inflammation (27, 28). Subsequently, this review expounds upon the potentiality of TCM in the realm of tumor immunotherapy, elucidating the prevailing clinical advancements in the manipulation via immune cells, modulation of cytokine signaling, angiogenesis and vascular normalization, extracellular matrix remodeling, inflammatory and immune responses, targeting immune checkpoints, and the modulation of immunosuppression and immune escape, respectively (**Figure 1**).

### 2.1 Modulation of immune cells

**Figure 2:**
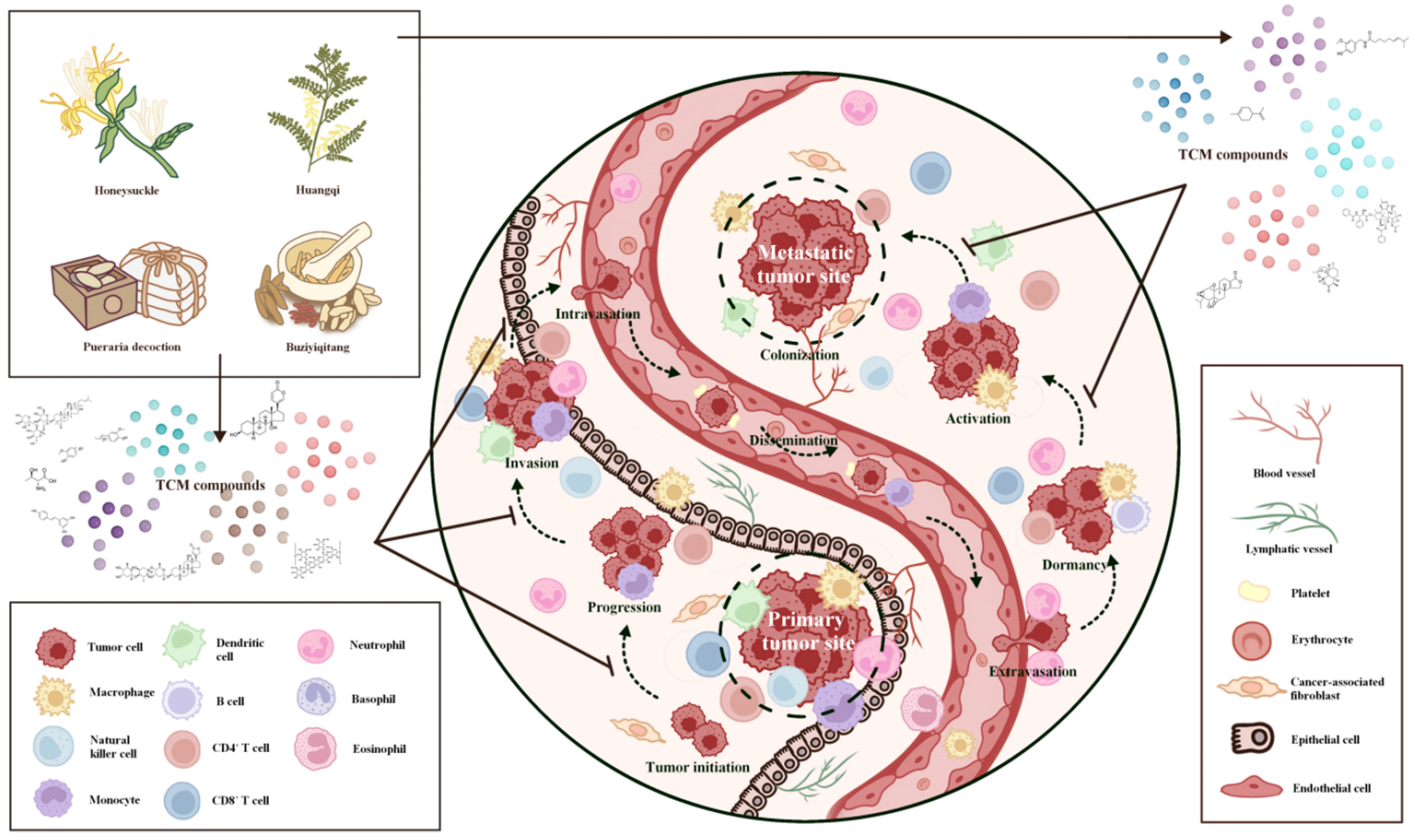
Inhibition of primary and metastatic tumor sites by virtue of traditional Chinese medicine-insights into its therapeutic potential. (Presented in the form of Taiji). TCM demonstrates a multifaceted impact on the suppression of primary and metastatic tumor sites. TCM restrains the growth and expansion of primary tumor cells by regulating cellular proliferation and apoptosis, impeding angiogenesis, and inhibiting tumor-promoting factors. Through its intricate network of bioactive compounds, TCM disrupts key signaling pathways implicated in tumor growth, progression, invasion, and metastasis, ultimately inhibiting tumor biological behaviors. TCM attenuates the formation of pre-metastatic niches by impeding the release of pro-metastatic factors, preventing the colonization and establishment of secondary tumor sites. By interfering with the epithelial-mesenchymal transition process, TCM hinders tumour cells from gaining invasive traits and reduces the likelihood of metastasis. By suppressing the invasive and migratory behavior of tumor cells, TCM curbs their ability to initiate metastasis and invade distant tissues. TCM exerts a modulatory effect on the tumor microenvironment, promoting immune surveillance and enhancing anti-tumor immune responses, which eventually hinders tumor development. These collective actions of TCM underscore its potential as an adjunctive therapy for combating primary and metastatic tumors, holding promise for the advancement of novel therapeutic strategies.

Tumor biological behaviors as a hot research topic have long been being investigated, including tumor initiation, progression, invasion, intravasation, dissemination, extravasation, dormancy, activation, and colonization. Immune cells matter a lot in affecting a series of tumor biological behaviors. Recently, TCMs have been reported to inhibit tumor biological behaviors via regulating immune cells. Relevant studies are introduced as follows and summarized in **Table 1** (29–46), and an elaborated graph is presented in the form of Taiji to demonstrate the interactions and relationships among tumor cells, immune cells and TCMs (**Figure 2**).

**Table 1:** mmunological intervention of TCM monomers and compound prescriptions on different cell types.

Cell Type	Medicine	Mechanism	Reference
**T cell**	Ginseng polysaccharide	Th1 cell-mediated anti-infection immune responses	(29)
	Ginsenoside Rg3	Increasing peripheral blood T lymphocyte subsets	(30)
	Bu-Zhong-Yi-Qi-Tang	Stimulating cytokines	(31)
	Quxie capsule	Increasing the fraction of CD8+ T cells	(32)
**B cell**	Ginseng	Enhancing B cell activity	(33)
	Gambogic acid	Inducing growth inhibition	(34)
	Angelica gigas polysaccharide	Boosting B cell immunogenicity	(35)
**Natural killer cell**	Ginseng	Enhancing NK92-MI cells activation	(36)
	Astragalus membranaceus	Upregulating IL-17D expression & inducing the aggregation of NK cells aggregation	(37)
	Sijunzi Tang	Decreasing PD-1/PD-L1 expression	(38)
	Gansui-Banxia Decoction	AKT/STAT3/ERK signaling pathway	(39)
**Macrophage**	Shuangshen Granule	Decreased TAMs & mTOR signalling inhibition	(40)
	Safflower polysaccharide	Through notch1 signaling pathway	(41, 42)
	Baicalin	Reducing M1-type polarization	(43)
	Angelica root	Inhibiting Stat3 phosphorylation	(44)
	Xiaoshui decoction	Enhancement of autophagy	(45)
**Dendritic cell**	Plantain	Triggering toll-like receptor 4	(46)

#### 2.1.1 T cells

T cells are a type of leukocytes that play an essential role in the immune system. T cells are responsible for recognizing and attacking foreign pathogens such as viruses, bacteria, and parasites, as well as abnormal or cancerous cells in the body (47). Based on their functions and surface markers, T cells can be categorized into various types, including helper T cells, cytotoxic T cells, regulatory T cells and memory T cells (48). TCM can intervene with T cell activity through various mechanisms. For instance, acidic polysaccharides in ginseng can induce the production of Th1 cells and macrophages, thus synergizing with recombinant interleukin-2 (rIL-2) to generate lymphokine-activated killer cells, potentially contributing to its anti-tumor mechanisms (49). Takei et al. discovered that ginsenosides can promote the transformation of initial T cells into typical Th1 cells, leading to the production of substantial amounts of IFN-γ and IL-4 (50). Additionally, research has indicated that baicalin may achieve the suppression of Th17 cells by reducing RORγt expression, upregulating Foxp3, and decreasing IL-17 expression. Moreover, baicalin can inhibit Th17 cell differentiation through reducing RORγt expression (51). Hence, baicalin holds promise as a therapeutic agent for Th17 cell-mediated diseases. In the tumor microenvironment, immunosuppressive factors secreted by tumor cells lead to increased frequency of CD4+Foxp3+ Tregs and MDSCs, which creates an immunosuppressive environment that reduces CD8 activity. By modulating the immunosuppressive state, Chinese herbal medicine fosters increased CD8+ T cell infiltration while exerting immunomodulatory effects on the tumor microenvironment, ultimately impeding tumor growth (18). For example, ginseng polysaccharide has demonstrated the ability to elevate interleukin-2 (IL-2), interleukin-8 (IL-8), and tumor necrosis factor-alpha (TNF-α) levels, activating Th1 cell-mediated anti-infection immune responses and enhancing immune regulation in the body (29). A meta-analysis has also shown that combining ginsenoside Rg3 with chemotherapy leads to increased peripheral blood T lymphocyte subsets, including CD3, CD4, CD8, and CD4/CD8, in non-small cell lung cancer (NSCLC) patients compared to chemotherapy alone, indicating improved immune system function in these patients (30). Moreover, a study by Liu at al. highlighted the immunomodulatory efficacy of the renowned TCM prescription, Bu-Zhong-Yi-Qi-Tang (BZYQT), which stimulates the production of several cytokines, including IL-2, IL-4, IL-5, and IFN-γ, from T lymphocytes (31). Furthermore, the Quxie capsule has shown its potential to impede intestinal tumorigenesis through the downregulation of pro-inflammatory cytokine expression and the reduction in the proportion of myeloid-derived suppressor cells (MDSCs), while concurrently increasing the fraction of CD8+ T cells within colon tumors (32). Hence, it can be inferred that individual constituents and compounds derived from TCM have the potential to modulate T cell immune function through diverse mechanisms, ultimately manifesting anti-tumor effects.

#### 2.1.2 B cells

B cells, a type of leukocytes, play a crucial role in the immune system by producing antibodies, which are proteins that aid in defending the body against infections. When a foreign pathogen enters the body, B cells recognize the pathogen’s unique antigens and produce antibodies that specifically target and neutralize the pathogen. Also, they are involved in the process of immunological memory, thus being essential for long-term immunity against many infectious diseases. Ginseng, a commonly used Chinese medicine, is believed to possess diverse pharmacological activities, including potential anti-tumor effects. Studies have indicated that ginseng can enhance B cell activity, promote antibody production, and enhance the function of the immune system (33). Shi et al. unearthed the remarkable potential of gambogic acid, a flavonoid compound extracted from the resin of the *Garcinia hanburyi*, in inducing growth inhibition and apoptosis in activated B-cell-like diffuse large B-cell lymphoma (DLBCL) tumor cells, both *in vitro* and *in vivo (* 34). Similarly, Kim et al. unveiled the immune-enhancing capabilities of *Angelica gigas* polysaccharides, which not only boost B-cell immunogenicity but also directly hamper tumor cell adhesion, thereby restraining tumor growth and metastasis (35). Although there are some studies supporting the relevance of TCM to the B cell-related pathway, these studies are still preliminary and more research is demanded to validate and further explore the mechanisms of action of TCM in the anti-tumor field.

#### 2.1.3 Natural killer cells

NK cells are important non-specific immune cells in the body. In the presence of tumor or virus-specific lgG antibodies, NK cells can specifically recognize and eliminate target cells bound to these IgG antibodies. Activated NK cells can also secrete cytokines such as IFN-γ, IL-2 and TNF to play an immunomodulatory role. Wang et al. reported that ginseng polysaccharide can effectively enhance the activation of NK92-MI cells, which then leads to the upregulation of receptors such as NKp30, NKp44, and NKp46, consequently improving the killing ability of NK92-MI cells by increasing the expression of key mediators like perforin and granzyme B (36). Furthermore, *Astragalus membranaceus* exhibits anti-tumor effects by upregulating IL-17D expression and inducing the aggregation of NK cells in the lung, which plays a vital role in the therapeutic approach for lung cancer (37). The efficacy of Sijunzi Tang, a classic TCM prescription, in impeding the proliferation of colon cancer cells has been substantiated through its ability to decrease the expression of PD-1/PD-L1 while augmenting the activity of NK cells (38). As reported by Feng et al., Gansui-Banxia Decoction (GSBXD) was observed to elevate the proportion of CD3- NK1.1+ NK cells, demonstrating its immunomodulatory potential by inhibiting the AKT/STAT3/ERK signaling pathway and suppressing IL-1β and IFN-γ (39). From the researches above, we can infer that TCM active compounds have a relatively close relationship with the activation of NK cells, then stimulating the natural immune system.

#### 2.1.4 Macrophages

Macrophages are multifunctional non-specific immune cells capable of phagocytosis and destruction of foreign bodies and self-dying cells. In the investigation involving K-Ras G12D spontaneous lung cancer model mice and Lewis lung cancer mice, the intervention of Shuangshen Granule (a combination of *Panax quinquefolius L*, *Cordyceps sinensis*, and *Panax notoginseng*) resulted in a notable reduction in subcutaneous tumor volume and a remarkable decrease in the proportion of F4/80+CD206+ tumor-associated macrophages (TAMs) (40). Further analyses on the genetic level also suggested the effects of Shuangshen Granule on immune microenvironment of lung cancer by modulating bone marrow differentiation through mTOR signalling inhibition (40). A study by Zhang et al. uncovered that safflower polysaccharide induced the polarization of macrophages to M1-type by activating the Notch1 signaling pathway to inhibit the invasion and metastasis of melanocytes (41). Similarly, Ando et al. observed that safflower polysaccharide induced the production of molecules associated with M1-type macrophage polarization in peritoneal macrophages of mice (42). Another study confirmed that baicalin could mitigate acute lung injury in mice by reducing M1-type polarization of U937 macrophages through the inhibition of HMGB-1 (23). Additionally, Angelica polysaccharide was found to suppress tumor metastasis by inhibiting Stat3 phosphorylation in macrophages and preventing their polarization toward the M2 phenotype (43). The TCM compound prescription, Xiaoshui decoction (XSD), has been documented to exhibit a notable capacity in stimulating the proliferation of M1 macrophages and diminishing the presence of M2 macrophages through the enhancement of autophagy. These findings signify the potential of XSD as a promising and innovative therapeutic option for integrated approaches targeting malignant pleural effusion (44). Accordingly, TCM can enhance immune activity by enhancing phagocytosis and killing of macrophages, thus playing the anti-tumor role.

#### 2.1.5 Dendritic cells

Dendritic cells (DCs) act as messengers between the innate and adaptive immune systems. They recognize and capture foreign antigens, such as bacteria, viruses, and tumor cells, and then present them to other immune cells, such as T cells and B cells (16, 45). Moreover, they help prevent the immune system from attacking the body’s own normal cells by presenting self-antigens to immune cells in a way that they do not stimulate an immune response (52). Studies have found that plantain, a perennial plant, plays a positive role in defending against viruses, inhibiting tumor cell proliferation, and enhancing immune function. In addition to whole Psyllium, the polysaccharide extracted from *Psyllium* is also one of the active components of immune regulation (46). Studies have revealed that the PLP-2 polysaccharide derived from *Plantago asiatica L*. can promote the phenotypic and functional maturation of dendritic cells and PLP-2 may activate the MAPK and NF-κB pathway by triggering toll-like receptor 4 on DCs (53, 54).

### 2.2 Modulation of cytokine signaling

Cytokines, essential molecular messengers that orchestrate immune responses, are targets of interest for modulating tumor immunity with TCM compounds. For instance, baicalein, an active ingredient extracted from *Scutellaria baicalensis*, was reported to play an anti-tumor role by regulating cytokine signaling pathway. It effectively inhibits tumor cell growth, induces apoptosis, and modulates immune cell activity. Notably, it inhibits IL-6 and STAT3 signaling pathways, thus impeding tumor growth and metastasis (55). Sini decoction (SND) is another widely used TCM known for its ability to suppress colorectal cancer liver metastasis and alleviate liver injury *in vivo (* 56). SND achieves this by upregulating IL-2 and IFN-γ and downregulating IL-10 and TGF-β (38). Another traditional prescription, Gansui-Banxia Decoction (GSBXD), created by sage Zhang Zhongjing, is employed to treat stagnation of evil heat and obstruction of qi. Studies by Feng et al. have demonstrated GSBXD’s antitumor immune activity by reducing the accumulation of MDSCs *in vivo*, possibly achieved through down-regulation of AKT/STAT3/ERK signaling pathways and suppression of IL-1β and IFN-γ (39). Wenjinghuoluo prescription, a TCM compound treatment of rheumatoid arthritis, was discovered to decrease the expression of pro-inflammatory cytokines (TNF-α, IL-1β, IL-6, and IL-17), thus effectively inhibiting bone erosion and osteophyte formation in joints (57). Considering the crucial role of cytokines in tumor occurrence and development, various Chinese medicines hold significant potential for development in regulating cytokine pathways to exert anti-tumor effects.

### 2.3 Modulation of angiogenesis and vascular normalization

Angiogenesis, the process of forming new blood vessels, is essential for tumor growth and metastasis. Emerging reports indicate that TCM compounds possess anti-angiogenic properties, inhibiting the abnormal and excessive blood vessel formation within the TME (58). These compounds specifically target key molecular pathways involved in angiogenesis, such as the vascular endothelial growth factor (VEGF) signaling. Additionally, TCM compounds have demonstrated the ability to induce vascular normalization, promoting the restoration of a more normal and functional vasculature within the tumor. This normalization can enhance drug delivery, immune cell infiltration, and overall therapeutic efficacy. Notably, β-Elemene stands out as it regulates the expression of crucial molecules implicated in tumor angiogenesis and metastasis, including VEGF, matrix metalloproteinases (MMPs), E-cadherin, N-cadherin, and vimentin (59). Another promising natural phytochemical is Ginsenoside Rg3, which has been extensively studied for its potential in cancer prevention and treatment. Its mechanisms of action encompass a wide range of anti-cancer activities, including apoptosis induction, inhibition of proliferation, metastasis, and angiogenesis, as well as boosting the immune response against cancer cells (30). In the quest for effective anti-angiogenic agents, researchers have looked into other natural compounds such as catechin gallate, astragaloside, and curcumin. These compounds have demonstrated their ability to effectively inhibit angiogenesis, thereby impeding the formation of new blood vessels that are crucial for tumor growth and metastasis (60). Curcumol, another natural substance, has shown promise in restraining cell growth and angiogenesis in non-small cell lung cancer (NSCLC). This potential is attributed to its ability to regulate the SP1/miR-125b-5p/VEGFA pathway, thus suppressing the expression of VEGF and limiting tumor blood vessel formation (61). Additionally, the compound menthol has been found to block the trans-activation of the transient receptor potential vanilloid 1 (TRPV1) by activating the transient receptor potential melastatin subtype 8 (TRPM8) ion channel. This activation inhibits VEGF-induced angiogenesis in specific cancer cells, such as uveal melanoma UM92.1 cells and PC-3 cells, providing a novel approach to target angiogenesis in cancer therapy (62–64). In conclusion, TCM compounds have shown anti-angiogenic properties by targeting key molecular pathways involved in angiogenesis, such as VEGF signaling. They can inhibit abnormal blood vessel formation within the TME and promote vascular normalization. This normalization enhances drug delivery, immune cell infiltration, and overall therapeutic efficacy.

### 2.4 Modulation of extracellular matrix remodeling

The extracellular matrix (ECM) within the TME plays a crucial role in providing structural support to tumor cells and exerting a significant influence on their behavior. Studies have demonstrated that TCM compounds possess the ability to regulate the intricate processes of ECM remodeling, including the degradation and deposition of ECM components. Notably, certain compounds exhibit compelling anti-fibrotic property, effectively inhibiting the excessive accumulation of collagen and other ECM proteins, which can otherwise impede the infiltration of immune cells (65). Furthermore, TCM compounds display the capacity to modulate the activity of MMPs, a group of enzymes involved in ECM degradation, thus exerting a consequential impact on tumor invasion and metastasis (66). For instance, a notable example is the Kushen injection compound, which has been corroborated to uniformly block the migration and invasion through EMC in colon, brain, and breast cancer cell lines (67). These findings underscore the potential of TCM in regulating the dynamic interactions between tumor cells and the ECM, suggesting their therapeutic relevance in impeding tumor progression and metastasis.

### 2.5 Modulation of inflammatory and immune responses

Inflammation and immune dysregulation within the TME contribute to tumor growth, immune evasion, and therapy resistance. TCM has been corroborated to exert immunomodulatory effects on tumor cells, influencing inflammatory and immune responses in the TME (68). For instance, extracts derived from *Curcuma longa* have demonstrated the capacity to modulate immune-inflammatory responses, offering a potential avenue for regulating the occurrence and progression of glioma (69). Furthermore, the extraordinary attributes of honeysuckle and Huangqi have been recognized in their ability to suppress pro-inflammatory cytokines, including IL-6 and TNF-α. These cytokines play pivotal roles in the initiation and progression of tumors (70). The presence of inflammation and immune dysregulation within the TME significantly contributes to tumor growth, immune evasion, and resistance to therapy.

### 2.6 Targeting immune checkpoints

Immune checkpoints are essential regulatory molecules that help maintain immune homeostasis. However, cancer cells can exploit these checkpoints to evade detection and destruction by the immune system, allowing tumors to evade immune surveillance (71). TCM compounds have shown promise in their ability to modulate immune checkpoint molecules, including PD-1, CTLA-4, and LAG-3 (72). One such example is the Gegen Qinlian decoction (GQD), a classical TCM formula. When used in combination with anti-mouse PD-1 treatment, GQD has been found to downregulate PD-1 expression and increase the levels of IL-2, effectively restoring T-cell functions by suppressing inhibitory checkpoints (73). Berberine (BBR), a well-known anti-inflammatory drug in TCM, has also been identified as a negative regulator of PD-L1 by researchers like Liu et al. Its administration has been shown to enhance the sensitivity of tumor cells to co-cultured T-cells by reducing PD-L1 levels in cancer cells (74). Additionally, Zhang et al. have reported that a compound known as CFF-1 exhibits tumor growth inhibition and prevents lung metastasis by blocking the PD-1/PD-L1 pathway. This action leads to an improved T lymphocyte immune response via the EGFR/JAK1/STAT3 pathway. As a result, CFF-1 shows promise as a potentially effective treatment to counteract tumor immunosuppression, particularly in prostate cancer patients (41). In short, TCM compounds have shown promise in modulating immune checkpoint molecules, such as PD-1, CTLA-4, and LAG-3, which are essential for maintaining immune homeostasis but can be exploited by tumors to evade immune surveillance.

### 2.7 Immunosuppression and immune escape

TME frequently demonstrates its immunosuppressive characteristics that enable tumors to evade immune surveillance. Extensive research has focused on exploring the potential of TCM compounds to counteract immunosuppression within the TME. One remarkable aspect of TCM compounds is their ability to modulate the activity of immunosuppressive cells, such as regulatory T cells (Tregs) and myeloid-derived suppressor cells (MDSCs). By targeting these cells, TCM compounds effectively reduce their negative impact on the anti-tumor immune response, creating a more conducive environment for fighting cancer (75). Moreover, TCM compounds have demonstrated the ability to inhibit the expression of immune checkpoint molecules, such as PD-1 and CTLA-4, thus reinstating immune cell functionality and facilitating robust tumor immune responses (60, 74, 76). In the pursuit of novel approaches, researchers have explored innovative strategies involving TCM compounds. For instance, co-encapsulation of drugs in nanoparticles has demonstrated encouraging chemo-immunotherapeutic effects in mice with hepatocellular carcinoma (HCC). This approach enhances survival rates without causing significant toxicity, pointing towards a potential breakthrough in cancer treatment (77). Furthermore, specific TCM compounds, like Feiji Recipe, have been identified for their ability to restore T cell functionality within the cancer microenvironment. In non-small cell lung cancer (NSCLC), Feiji Recipe interferes with the indoleamine-2,3-dioxygenase pathway, a critical mediator of immune escape, offering a potential therapeutic avenue to counteract immune evasion in this type of cancer (78). By effectively addressing immunosuppression and reinvigorating the anti-tumor immune response, TCM-based treatments offer a potential complement or alternative to conventional therapies.

## 3 New findings of TCM natural products and derivatives as immunomodulating agents in cancer treatment

Numerous studies have provided evidence supporting the multifaceted potential of TCM. These properties encompass antibacterial, antioxidant, anti-inflammatory, anti-proliferative, anti-cancer, and various other beneficial effects. In this part, pertinent studies on several typical TCM natural products that have potential anti-cancer property are expounded in the following paragraphs and summarized in **Table 2** (39, 79, 80, 82–124, 132). Additionally, some closed and ongoing national clinical trials (NCT) on the association between TCMs and multiple cancer types are also displayed in **Table 3**.

**Table 2:** The summary table of several significant anti-tumor TCMs.

Category	Drug name	Structure	Main function	Target	Pathway	Reference
Polyphenols	*Curcumin* (PubChem CID: 969516)	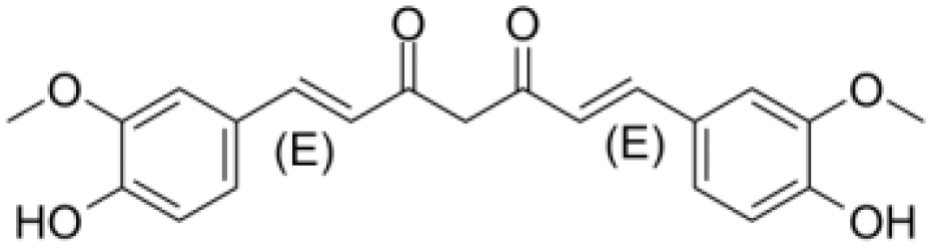	Antioxidant and anti-inflammatory	NF-κB; COX-2; TNF-α	1)NF-κB pathway; 2)MAPKs pathway; 3)PI3K/Akt pathway; 4)Wnt/β-catenin pathway; 5)Notch pathway	(79–81)
	*Resveratrol (Rsv)* (PubChem CID: 445154)	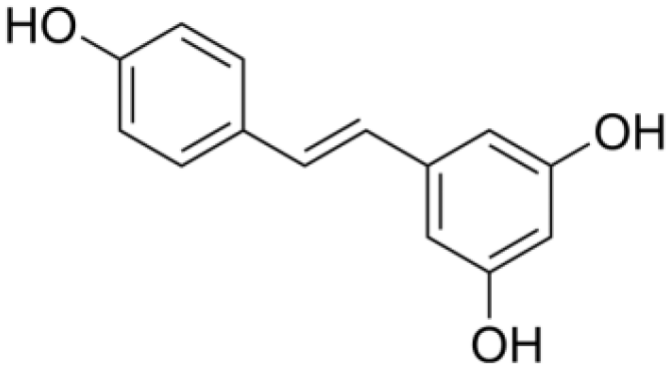	Antioxidant and anti-inflammatory	IKKβ; autophagy; mitophagy	1)autophagy pathways; 2)NF-κB pathway	(82–84)
Cardiotonic steroids	*Digoxin* (PubChem CID: 2724385)	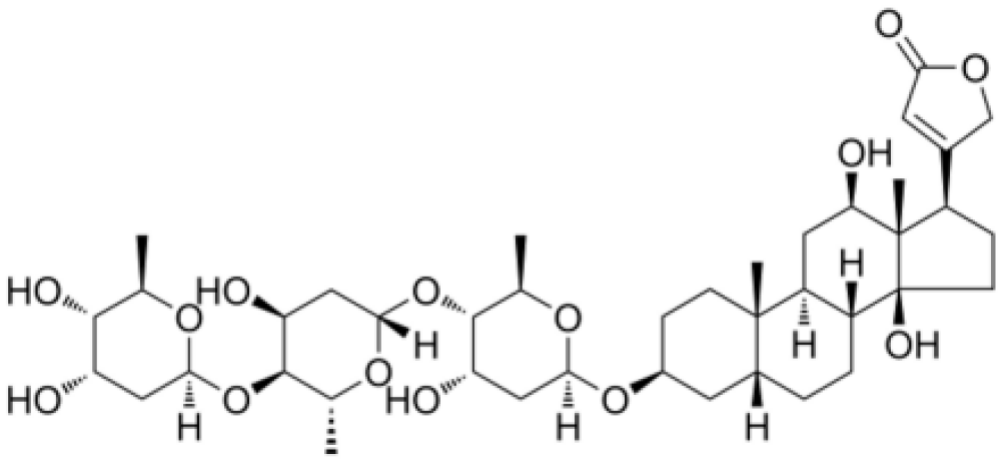	Positive inotropic action	Na+/K+ ATPase	Na+/K+ ATPase	(85–88)
	*Bufalin* (PubChem CID: 9547215)	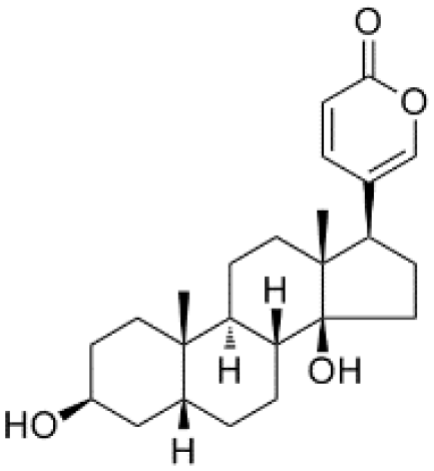	Cardiotonic, anti-inflammatory, and cancer suppressive	Na+/K+ ATPaseα3	c-Myc/NF-κB pathway	(89–93)
Terpenoids	*Limonene* (PubChem CID: 22311)	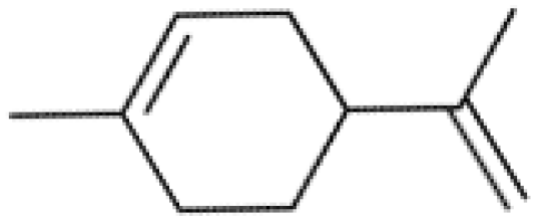	Antibacterial, anti-proliferative, antioxidant, and anti-inflammatory	TRP3; TRP4	1)Ras pathway; 2)Akt/mTOR pathway	(94–96)
	*Paclitaxel* (PubChem CID: 36314)	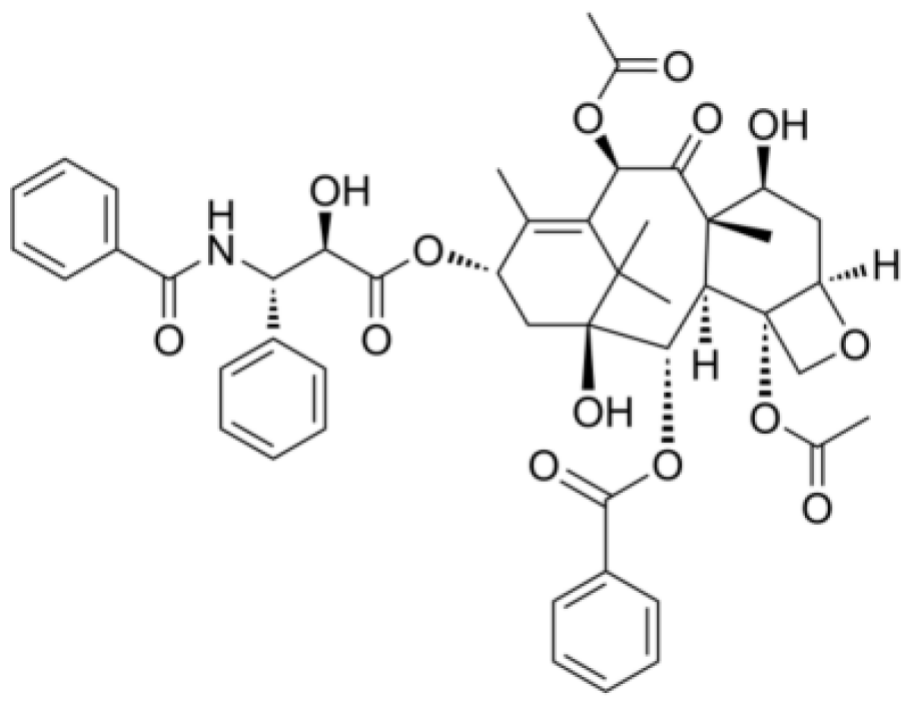	Stabilizing microtubules, inducing cell cycle arrest and inhibition of cell division	Microtubule protein; tubulin	1)antibody-drug conjugate: ADC cytotoxin; 2)cytoskeleton: microtubules/tubulin; 3)mitotic checkpoint pathway; 4)apoptotic pathways	(97, 98)
	*Artemisinin* (PubChem CID: 68827)	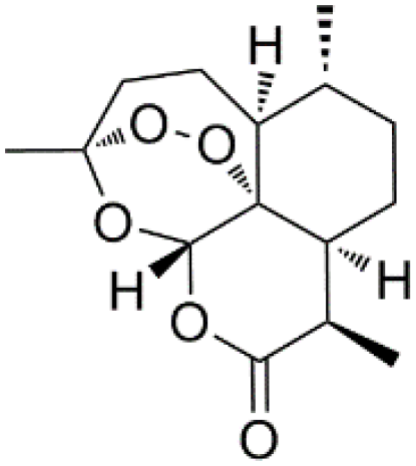	Antimalarial activity	Plasmodium falciparum ATPase 6	Heme detoxification pathway	(99–101)
	*Triptolide* (PubChem CID: 107985)	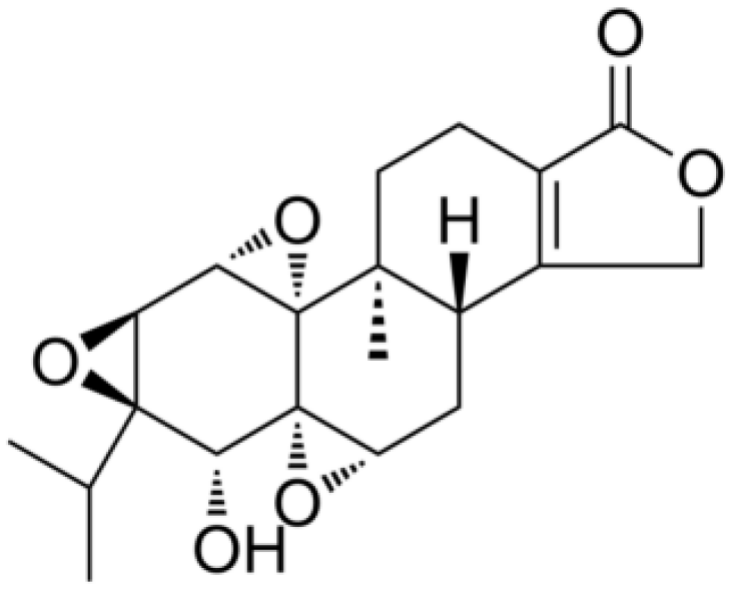	Immunosuppressive, anti-inflammatory, and anti-proliferative	HSP90; NF-κB; MDM-2/p53	1)NF-κB pathway; 2)JAK-STAT pathway; 3)Wnt/β-catenin pathway	(102–108)
	*Polysaccharides* (PubChem SID: 405235487)	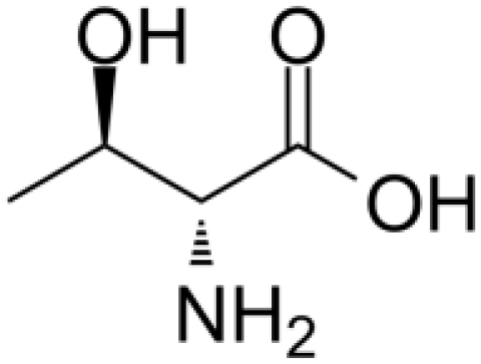	Immunomodulatory, anti-inflammatory, and antioxidant effects	MMP-2; MMP-9; ERK1; ERK2	1)MAPK/ERK pathway; 2)JNK pathway	(109–111)
Polvsaccharides	*Lentinan* (PubChem CID: 37723)	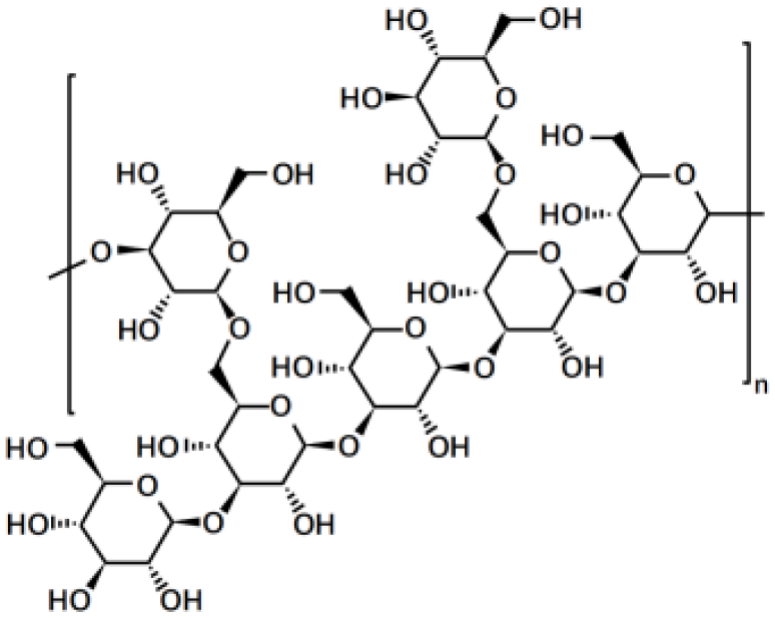	Immunomodulatory activity	Dectin-1; TLR2; TLR4	1)NF-κB pathway; 2)MAPK pathway	(112–115)
Saponins	*Panax notoginseng Saponins* (PubChem CID: 297)	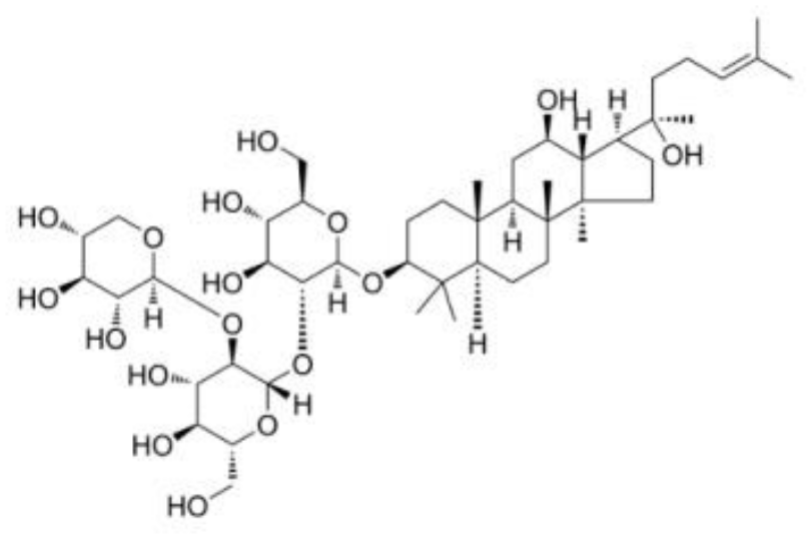	Influencing cardiovascular function, inflammation, and oxidative stress	Endothelial nitric oxide synthase (eNOS)	1)PI3K/Akt pathway; 2)ERK pathway	(39, 116–118)
Capsaicin	*Capsaicin* (PubChem CID: 1548943)	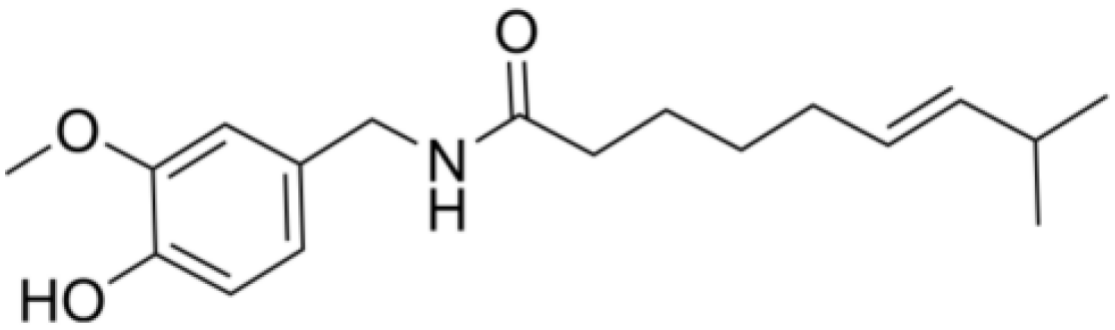	Interacting with sensory neurons (TRPV1) ion channel; anti-inflammatory	TRPV1; neutrophils; macrophages	1)MAPK pathway; 2)NF-κB pathway	(119–124)

**Table 3:** Closed and ongoing national clinical trials (NCT) on the association between TCMs and multiple cancer types.

Clinical trial. gov identifier	Official title	Model	Conditions	Intervention/Treatment	Enrollment/Estimated enrollment	Primary outcome	Completion date/Estimated completion date
NCT01780181	Randomized Double-blind Controlled Clinical Study of Chemotherapy Combined With or Without Traditional Chinese Medicine on Survival Effect of Elderly Patients With Advanced Non-small-cell Lung Cancer	Case-control	Lung cancer	TCM	82	Progression free survival	2016/09/01
NCT01745302	Study of Chinese Medicine Plus EGFR-TKI Versus EGFR-TKI in Advanced Pulmonary Adenocarcinoma: a Randomized Double-blind Controlled Clinical Trial	Case-control	Pulmonary adenocarcinoma	TCM	470	Progression free survival	2016/12/01
NCT03332368	Clinical Study on Prevention and Treatment of Recurrent and Metastasis of Triple Negative Breast Cancer(TNBC) With Traditional Chinese Medicine(TCM)	Cohort	Pulmonary adenocarcinoma	TCM	620	Progression free survival; overall survival	2019/01/01
NCT03607656	The Effect of Traditional Chinese Treatment Combined Adjuvant Chemotherapy in IIIb and IIIc Gastric Cancer: A Randomized Controlled Trial	Parallel assignment	Gastric cancer	Oxaliplatin	270	3-year disease free survival rate	2023/12/31
NCT02889692	Study of Chinese Medicine Plus Targeted Therapy Maintenance Versus Targeted Therapy Maintenance in Advanced Pulmonary Adenocarcinoma: A Randomized Double-blind Controlled Clinical Trial	Parallel assignment	Pulmonary adenocarcinoma	TCM	23	Overall survival	2017/06/01
NCT04482829	Efficacy and Safety of Jing-yuan-kang Granule in the Treatment of Lung Adenocarcinoma	Parallel assignment	Lung adenocarcinoma	Jing-yan-kang granule	144	1) The quality of life will be evaluated by the European Organization for Research and Treatment of Cancer Quality of life Questionnaire Core-30 (EORTC QLQ-C30) scale; 2) Quality of life will be evaluated by Quality of life Questionnaire Lung Cancer-13 (QLQ-LC13)	2022/06/30
NCT01975454	A Pilot Study of Teng-Long-Bu-Zhong-Tang Based Herbal Therapy in Combination With Chemotherapy in Patients With Metastatic Colorectal Cancer	Parallel assignment	Metastatic colorectal cancer	Herbal therapy	62	Progression free survival	2017/12/01
NCT02929693	Clinical Study of Yiqi-yangyin-jiedu Decoction Combined With Gefitinib in Advanced Pulmonary Adenocarcinoma Patients With Activating EGFR Mutation	Parallel assignment	Pulmonary adenocarcinoma	Yiqi-yangyin-jiedu decoction	198	Progression free survival	2019/06/01
NCT04438564	Immunoassay and Regulation of Traditional Chinese Medicine on Cancer Patients	Single group assignment	breast Cancer	TCM	300	The IL-2, TNF-α and IFN-γ expression by TCM treatment on cancer patients	2023/07/31
NCT05894694	Survival Benefit of Compound Kushen Injection in Treatment of Advanced Colorectal Cancer Based on Real World Registration Platform	Parallel assignment	Colorectal carcinoma	Kushen injection	318	Progression free survival	2024/12/30
NCT00094445	Phase II Trial of Curcumin in Patients With Advanced Pancreatic Cancer	Single group assignment	Pancreatic neoplasms	Curcumin	50	Six-month participant survival	2014/04/01
NCT03980509	A “Window Trial” on Curcumin, the Active Compound in Turmeric, for Invasive Breast Cancer Primary Tumors	Single group assignment	Breast cancer	Curcumin	22	Changes in tumor proliferation rate	2023/08/31
NCT03769766	A Randomized, Double-Blind, Placebo-Controlled Trial of Curcumin to Prevent Progression of Biopsy Proven, Low-risk Localized Prostate Cancer Patients Undergoing Active Surveillance	Parallel assignment	Prostate cancer	Curcumin	291	Rate of disease progression	2026/11/01
NCT01476592	A Biological Study of Resveratrol’s Effects on Notch-1 Signaling in Subjects With Low Grade Gastrointestinal Tumors	Single group assignment	Gastrointestinal tumors	Resveratrol	7	Notch1 activation in post-treatment tumor biopsy specimens when compared to pretreatment levels	2018/10/11
NCT00256334	Resveratrol for Patients With Colon Cancer	Single group assignment	Colon cancer	Resveratrol	11	Test the hypothesis that resveratrol modulates Wnt signaling *in vivo* in colon cancer and normal colonic mucosa	2009/04/01
NCT01162135	A Pilot Phase II Study of Digoxin in Patients With Recurrent Prostate Cancer as Evident by a Rising PSA	Single group assignment	Prostate cancer	Digoxin	16	Rate of positive PSADT outcome	2013/05/01
NCT01046929	Clinical Study of Limonene in Women With a Recent Diagnosis of Early Stage Breast Cancer Electing to Undergo Excision Surgery	Single group assignment	Breast cancer	Limonene	59	Breast tissue limonene level	2011/03/01
NCT00002917	A Phase I/II Institutional Study of Intravesical Taxol (Paclitaxel) Instillation for the Treatment of Superficial Bladder Cancer	Not provided	Bladder cancer	Paclitaxel	19	Not provided	2004/05/01
NCT00989131	An Open, Randomized, Multicenter Study in Patients With Recurrent Epithelian Cancer, Primary Peritoneal Cancer or Fallopian Tube Cancer to Compare the Efficacy and Safety of Paclitaxel (Micellar) Nanoparticles and Paclitaxel (Cremophor^®^ EL)	Parallel assignment	Epithelial ovarian cancer	Paclical	789	1) Progression free survival; 2) Changes in area under the curve of CA-125; 3) Incidence and severity of hypersensitivity reactions	2013/10/01
NCT00764036	Prospective Open Uncontrolled Phase I Study of Compatibility, Safety&Pharmacokinetics of Artesunate, a Semisynthetic Derivative of Artemisinin From the Chinese Herb Artemisia Annua in Patients With Metastatic/Locally Advanced Breast Cancer	Sequential assignment	Breast Cancer	Artesunate	23	Dose limiting adverse events with possible, probable or definite relation with the respective dose level of the add-on therapy	2013/11/01

*Polyphenols* represent a class of naturally occurring compounds abundantly found in multiple plants, encompassing fruits, vegetables, and herbs. These molecules possess distinctive chemical and biological properties owing to their multiple phenol rings. Research has consistently revealed the wide-ranging health benefits of polyphenols, including their remarkable anti-inflammatory, antioxidant, and anti-cancer effects (125, 126). These compounds have been observed to exert modulatory influences on a series of cellular pathways critical to disease development and progression, such as cell cycle regulation, apoptosis, and angiogenesis (125, 127). *Curcumin*, derived from the dried rhizome of turmeric, is rich in phenols, terpenoids, flavonoids, and other constituents. Specifically categorized as a diphenyl heptane within the phenolic group, curcumin treatment has been discovered to effectively impede the proliferation and promote the apoptosis of colon cancer cells (128). Notably, curcumin treatment elevates the expression of miR-206, which subsequently influences the functional behavior of colon cancer cells. Via augmenting colon cancer cell apoptosis and suppressing PD-L1 expression, miR-206 enhances the cytotoxic effects of T cells on tumor cells. This potentiation is achieved through the inhibition of PD-L1 via suppression of the JAK/STAT3 pathway (57). These compounds, with their distinctive phenol rings, have been consistently shedding light on new horizons in cancer immunotherapy.

*Resveratrol (Rsv)*, another prominent polyphenol compound, is predominantly sourced from plants such as peanuts, grapes, *Polygonum cuspidatum*, and mulberries (82). Resveratrol exhibits a multitude of pharmacological property, notably its antioxidant, anti-inflammatory, and anti-cancer activities, which render it highly beneficial for the treatment of gastric diseases (83). Resveratrol’s mechanisms of action involve modulating the SLC7A11-HMMR interaction, activating ferroptosis, enhancing CD8+ T cell cytotoxicity, and regulating the tumor immune microenvironment (82). In summary, polyphenols offer a promising avenue in the field of health and disease management, with their diverse chemical properties and potential effects on essential cellular pathways. The specific polyphenols curcumin and resveratrol have shown remarkable impacts on cancer cells and the immune microenvironment, holding significant potential for therapeutic applications.

*Cardiac glycosides*, natural sterols derived from plants and animals, are known for their cardiotonic properties, enhancing cardiac contractions by targeting the cellular sodium-potassium ATPase pump (85). Extensive evidence suggests the remarkable antitumor property of cardiac glycosides, making them highly recommended for the treatment of diverse cancer types (85, 129, 130). Moreover, mutated or dysregulated transcription factors have emerged as promising therapeutic targets amenable to selective intervention. *Neriifolin*, a cardiac glycoside, has demonstrated its potential in suppressing malignant characteristics of prostate cancer cells. It achieves this by activating endoplasmic reticulum stress (ERS), which impacts the induction of antitumor inhibitory receptors, thereby enhancing DNA damage and apoptosis (131). *Digoxin*, a classic cardiac glycoside, exhibits potential in reducing inflammation and fibrosis in a mouse model of pulmonary fibrosis. Its mechanism involves inhibiting the mTORC2/AKT/IRF4 signaling pathway and macrophage M2 polarization (86). *Bufalin*, a major component of the dried secretion of Bufo, represents another cardiac steroid widely employed for therapeutic purposes. *Bufalin* exerts anti-breast cancer effects through multiple pathways and targets. It inhibits the PI3K/AKT pathway, induces apoptosis via the mitochondrial pathway, triggers programmed necrosis through the RIP11/RIP3/PARP-1 signaling pathway, and hinders cell proliferation and invasion by influencing cell cycle progression and inhibiting epithelial-mesenchymal transition (EMT) (89, 90, 132). In summation, cardiac glycosides have etched their mark not only in the realm of cardiotonic therapy but also as a burgeoning frontier in the battle against cancer.

*Terpenoids*, an expansive and diverse class of organic compounds prevalent in numerous plants, including cannabis, contribute significantly to the distinctive aroma and flavor of various botanical specimens. Additionally, terpenoids are renowned for their remarkable medicinal property, with several of them demonstrating potent anti-cancer effects. One such terpenoid, *Thymol*, can induce an anticancer effect in acute promyelocytic leukemia cells by arresting the cell cycle at G0/G1 phase and mitochondrial depolarization through reactive oxygen species production followed by mitochondrial membrane deterioration (133). Another noteworthy terpenoid, *limonene*, abundantly found in citrus fruits and specific cannabis strains, has shown anti-cancer effects by inducing apoptosis in breast cancer cells, promoting programmed cell death in the cancerous cells (94). *Paclitaxel*, a chemotherapy drug derived from the *Pacific yew* tree, hinders cancer cell division and growth by effectively inhibiting the breakdown of microtubules, cellular structures vital for maintaining cell shape and facilitating cell division. By binding to microtubules, *paclitaxel* prevents their disassembly, thereby thwarting the ability of cancer cells to divide and proliferate (97). *Artemisinin*, a compound derived from the sweet wormwood plant and long utilized in TCM for malaria treatment, has recently been discovered to possess anti-cancer property by generating free radicals within cancer cells, leading to oxidative stress and subsequent cell death. *Artemisinin* has exhibited notable efficacy against breast cancer cells (99, 100). *Triptolide*, derived from the thunder god vine plant and traditionally employed in Chinese medicine to address many conditions, including inflammation and autoimmune diseases, has also demonstrated potent anti-cancer property inhibiting the NF-kB protein, a crucial regulator of cell survival, and inducing apoptosis in cancer cells (102–104).

*Polysaccharides*, intricate carbohydrates abundant in plants, fungi, and bacteria, exhibit a diverse array of health benefits, including their remarkable anti-cancer property. Extensive research has demonstrated that polysaccharides possess the ability to impede the growth and proliferation of cancer cells through activating the immune system and promoting the production of cytokines. After the immune system is activated, polysaccharides facilitate the identification and elimination of cancer cells (109). Moreover, polysaccharides showcase direct anti-cancer effects in addition to their immune-boosting attributes. Notably, polysaccharides derived from mushrooms have demonstrated desirable efficacy in inhibiting cancer cell growth by inducing apoptosis or programmed cell death. Furthermore, by inducing apoptosis and inhibiting the formation of blood vessels crucial for cancer cell proliferation and metastasis, mushroom polysaccharides demonstrate potent anti-cancer efficacy (29, 109, 110). For instance, *lentinan*, a polysaccharide extracted from the shiitake mushroom, has garnered significant attention for its anti-cancer potential. It effectively hinders cancer cell growth and proliferation by activating the immune system and stimulating cytokine production, thus enhancing immune responses against cancer cells (112, 134). Clinical trials evaluating lentinan have shown promising therapeutic capabilities, particularly in gastric cancer and colorectal cancer. Patients treated with lentinan experienced improved survival rates and enhanced quality of life, as the polysaccharide not only combats cancer cells but also alleviates the side effects of chemotherapy and radiation therapy (112). In essence, polysaccharides, with their immune-activating and direct anti-cancer attributes, stand at the forefront of the evolving landscape of cancer therapy.

*Saponins*, a group of plant glycosides with their distinctive ability to generate abundant foam in aqueous solutions, have garnered attention due to their profound anti-cancer effects (116, 135). These compounds effectively impede cancer cell growth and proliferation by inducing apoptosis. In Chen’s clinical trial, a significant discovery was made regarding the therapeutic potential of combining ginsenoside Rg3 with MF chemotherapy. The findings revealed a notable reduction in serum VEGF concentration, resulting in improved survival rates for patients with advanced gastric cancer (136). Another notable saponin, *Saikosaponin D*, demonstrated impressive efficacy in enhancing apoptosis mediated by TNF-α in HepG2 cells. This effect was attributed to the inhibition of NF-κB activation and downregulation of target genes associated with cancer cell proliferation, angiogenesis, invasion, and survival (137). Notably, ginsenoside Rg-3 has recently gained recognition in China as a promising anti-angiogenic and anti-cancer drug (116). By activating the immune system and stimulating cytokine production, saponins can identify and suppress the activity of cancer cells. *Panax notoginseng Saponins (PNS)* have shown cytotoxicity and enhanced chemosensitivity in pancreatic cancer cells treated with Gemcitabine. This effect was achieved through the inhibition of autophagy and promotion of apoptosis, providing a potential treatment option for pancreatic cancer (116). These compounds, by effectively curbing cancer cell growth and proliferation through apoptosis induction, offer a compelling avenue for exploring innovative cancer therapeutics.

*Capsaicin*, a vanilloid phytochemical found in chili peppers and pepper extracts, exhibits significant anti-cancer properties against various human cancers, including lung, gastric, colon, and breast cancer. Its anti-cancer effects are attributed to its ability to induce apoptosis and inhibit tumor cell proliferation. Additionally, capsaicin exhibits noteworthy anti-inflammatory property, which contribute to its cancer-preventive potential by mitigating chronic inflammation. In specific cancer types, capsaicin has been observed to induce apoptosis in ORL-48 cells through the intrinsic apoptotic pathway, involving disruption of mitochondrial membrane potential and activation of caspase-3, -7, and -9 (138). In bladder cancer cells, capsaicin downregulates tNOX expression, leading to enhanced apoptosis and reduced cell growth. The inhibition of extracellular regulated protein kinases activation by capsaicin results in decreased phosphorylation of paxillin and focal adhesion kinase (FAK), leading to reduced cell migration (119). Thoennissen et al. found that in breast cancer cells, capsaicin modulates the EGFR/HER-2 pathway, suggesting its potential role in breast cancer treatment and prevention (120). Furthermore, Jin et al. reported capsaicin’s profound anti-proliferative effects on colon cancer cells involve cell cycle arrest in the G0/G1 phase and promotion of apoptosis, associated with increased expression of p21, Bax, and cleaved poly ADP-ribose polymerase (PAARP), which play essential roles in regulating cell cycle progression and apoptosis (121). In conclusion, *capsaicin*, whose impact reverberates across various human cancers, from lung and gastric to colon and breast cancer, offers a promising avenue for novel cancer interventions.

## 4 Future perspectives

Tumor immunotherapy can activate the immune system to identify and eliminate cancer cells. Accordingly, cancer cells produce suppressive proteins to alleviate immune responses and evade immune surveillance. Immunotherapy agents possess the capability to block these proteins, thereby facilitating the immune system’s efficient recognition and elimination of cancer cells (5, 129, 139). The paramount significance of tumor immunotherapy stems from its capability to harness the formidable potential of the immune system to destroy cancer cells. Diverging from conventional cancer treatments like chemotherapy and radiation therapy, which indiscriminately harm both normal and cancerous cells, immunotherapy selectively targets cancer cells and spares normal cells from harm. Consequently, this approach holds promise as a more potent and less toxic treatment modality for cancer patients (140). Additionally, immunotherapy has displayed promising outcomes in the treatment of multiple cancer types, including melanoma, lung cancer, and bladder cancer (141, 142). It has also demonstrated desirable efficacy in treating cancers that have developed resistance to conventional therapeutic interventions.

Despite its considerable potential benefits, tumor immunotherapy is accompanied by several limitations that necessitate attention and resolution. One primary limitation is the heterogeneous response observed among patients undergoing immunotherapy (143). While some individuals have exhibited remarkable and favorable responses, others have not experienced any discernible benefits. This heterogeneity may arise from variances in patients’ immune system profiles or the distinct characteristics of their specific cancers. Another limitation of immunotherapy pertains to the potential occurrence of side effects (144). Although immunotherapy generally exhibits lower toxicity compared to traditional cancer treatments, it can still induce immune-related side effects such as inflammation, fatigue, and cutaneous manifestations (144, 145). In certain instances, these side effects may severely manifest and urgently demand intervention. The third limitation is its relatively high expenditure. Immunotherapy drugs often incur greater expenses than conventional cancer treatments, thereby posing a financial barrier to access for some cancer patients with low income.

TCM harbors notable potential in the realm of tumor immunity for several compelling reasons. Acknowledging the interconnected nature of the immune system with other bodily systems, TCM endeavors to rectify underlying imbalances, thus enhancing the body’s inherent defense mechanisms, including tumor immunity (146). Secondly, TCM compounds have exhibited immunomodulatory property, influencing the function of immune cells, cytokine signaling, and immune responses. These compounds possess the capacity to augment the activity of immune cells crucial for tumor immunity, while concurrently suppressing immunosuppressive cell populations. Through modulating the immune response, TCM compounds are promising in promoting anti-tumor immune responses and bolstering tumor eradication. They can concurrently target multiple immune checkpoints, cytokines, and cellular pathways, culminating in a comprehensive and multi-faceted approach that augments tumor immunity. Such multimodal effects hold the potential to surmount the limitations associated with therapies focusing on a single target and subsequently enhance therapeutic outcomes. Furthermore, TCM places considerable emphasis on personalized medicine, tailoring treatments to address the specific requirements and imbalances exhibited by each individual patient (147). This personalized approach acknowledges the inherent variability of tumor immunity among patients, attributable to factors such as genetic predisposition, immune status, and the distinctive characteristics of the tumor microenvironment. Lastly, TCM boasts a rich historical heritage, spanning centuries of utilization in China and other Asian nations, thereby accumulating extensive clinical experience in managing a diverse range of health conditions. Traditional herbal formulations and treatment methodologies have been refined over time based on empirical observations and accumulated wisdom. This fusion of historical knowledge with modern scientific investigations presents a distinctive opportunity to harness the potential of TCM in optimizing tumor immunity.

TCM encompasses an extensive repertoire of herbal compounds, possessing infinite potential for immunomodulation. One avenue of progress involves the identification and isolation of specific active compounds present in TCM formulations that exert robust effects on tumor immunity. By isolating these compounds and subjecting them to rigorous scientific scrutiny, their mechanisms of action can be elucidated, enabling a more precise evaluation of their efficacy and safety. Further investigations are warranted to comprehend the underlying mechanisms by which TCM compounds modulate tumor immunity. This necessitates exploring the signaling pathways, cellular interactions, and molecular targets involved in the immunomodulatory effects of TCM compounds. Mechanistic studies offer invaluable insights into the precise actions of TCM compounds on immune cells, cytokine signaling, immune checkpoints, and the tumor microenvironment, thereby guiding the advancement of targeted therapeutic interventions. Given the complexity and heterogeneity of tumor immunity, which varies among individuals and tumor types (148), the development of personalized TCM treatment strategies hinges on the identification of biomarkers capable of predicting treatment responses and patient outcomes. The identification of such biomarkers can facilitate the selection of TCM compounds and formulations tailored to individual patients, optimizing their potential to enhance tumor immunity and improve treatment efficacy (149). By steadfastly pursuing the above exploratory directions, TCM can continuously evolve as a valuable approach for augmenting tumor immunity, ultimately contributing to the advancement of more efficacious and personalized strategies for cancer treatment.

The emergence of precision medicine has fundamentally transformed the landscape of cancer treatment, revolutionizing therapeutic approaches by tailoring interventions to individual patients based on their unique genetic and molecular characteristics (150). In this context, TCM emerges as a highly promising complementary modality, capable of augmenting the efficacy and personalization of cancer immunotherapy. Through the identification of specific TCM compounds and formulations that align with individual tumor and immune profiles, clinicians are more likely to optimize treatment strategies, enhance therapeutic outcomes, and minimize adverse effects. Moreover, the integration of TCM with conventional cancer therapies like chemotherapy and radiation, paves the way for synergistic effects and novel therapeutic avenues. Notably, studies have indicated that TCM compounds can enhance the effectiveness of these treatments by sensitizing tumor cells to cytotoxic agents, modulating the tumor microenvironment, and mitigating treatment-associated side effects (72). Furthermore, the combination of TCM with emerging immunotherapies, such as cancer vaccines and chimeric antigen receptor (CAR) T-cell therapy, embraces a promising future in unleashing robust anti-tumor immune responses and ultimately improving patient outcomes. Finally, the combination of traditional wisdom and modern scientific advancements assumes paramount importance in propelling the progress of TCM within the realm of cancer immunotherapy (151). To this end, rigorous scientific investigations on preclinical and clinical studies are indispensable for elucidating the mechanisms of action, validating efficacy, and ensuring the safety of TCM compounds.

## 5 Conclusion

The potential of TCM in the realm of anti-cancer immunotherapy is supposed to be cherished, and its immunoregulatory mechanisms should be shed light on. This review summarizes the current state of research, also categorizing and expounding broadly applied TCM approaches that possess anti-cancer property. Additionally, the curative effects and underlying mechanisms of several frequently used Chinese medicines are discussed meticulously. Moreover, the mechanism of several commonly used TCM monomers and compound prescription were explained in detail. Overall, our work presents a comprehensive overview of the progress made in TCM for cancer treatment, providing valuable insights into its advancement in this field.

## Author contributions

KM: Resources, Visualization, Writing – original draft. WL: Data curation, Visualization, Writing – original draft. JX: Visualization, Writing – review & editing. ZQ: Conceptualization, Resources, Project administration, Visualization, Writing – review & editing. QZ: Conceptualization, Funding acquisition, Project administration, Visualization, Writing – review & editing.
